# Internações por aborto no Brasil, 2008-2018: estudo ecológico de série temporal

**DOI:** 10.1590/S1679-49742022000100017

**Published:** 2022-02-28

**Authors:** Maíra Dutra Uliana, Daniela Ferreira D’Agostini Marin, Maura Belomé da Silva, Camila Giugliani, Betine Pinto Moehlecke Iser

**Affiliations:** 1 Universidade do Sul de Santa Catarina, Faculdade de Medicina, Tubarão, SC, Brasil Universidade do Sul de Santa Catarina Universidade do Sul de Santa Catarina Faculdade de Medicina Tubarão SC Brazil; 2 Universidade Federal do Rio Grande do Sul, Programa de Pós-Graduação em Epidemiologia, Porto Alegre, RS, Brasil Universidade Federal do Rio Grande do Sul Universidade Federal do Rio Grande do Sul Programa de Pós-Graduação em Epidemiologia Porto Alegre RS Brazil; 3 Universidade do Sul de Santa Catarina, Programa de Pós-Graduação em Ciências da Saúde, Tubarão, SC, Brasil Universidade do Sul de Santa Catarina Universidade do Sul de Santa Catarina Programa de Pós-Graduação em Ciências da Saúde Tubarão SC Brazil

**Keywords:** Aborto, Hospitalização, Saúde da Mulher, Saúde Pública, Estudos de Séries Temporais, Abortion, Hospitalization, Women’s Health, Public Health, Time Series Studies, Aborto, Hospitalización, Salud de la Mujer, Salud Pública, Estudios de Series Temporales

## Abstract

**Objetivo:**

Analisar a tendência temporal das internações por aborto no Brasil, de 2008 a 2018, segundo região e Unidades da Federação (UFs).

**Métodos:**

Estudo ecológico, com dados de internações por aborto de mulheres em idade fértil registrados no Sistema de Informações Hospitalares/Sistema Único de Saúde (SIH/SUS). As taxas foram calculadas segundo características da mulher; e a tendência, avaliada por regressão linear generalizada de Prais-Winsten.

**Resultados:**

As 2.258.104 internações por aborto representaram 5% de todas as internações de mulheres em idade fértil. Houve redução significativa, de 0,76 pontos percentuais ao ano, no período. Essa tendência ocorreu em 19 UFs brasileiras e em todas as regiões, exceto a Sul (estável). Houve redução significativa (p-valor<0,001) nas internações por aborto espontâneo e nas internações de mulheres de 20 a 39 anos.

**Conclusão:**

Observou-se tendência de redução das internações por aborto no país, com variações segundo características da mulher, UF e região de residência.

## Abstract (en)

**Objective:**

To analyze the temporal trend of hospitalizations caused by abortion in Brazil between 2008-2018, according to region and federal units (FU).

**Methods:**

Ecological study concerning hospitalizations due to abortion of women of childbearing age, from Hospital Information System data. The rates were calculated according the characteristics of women. The trend was assessed by Prais-Winsten generalized linear regression.

**Results:**

The 2,258,104 hospitalizations due to abortion represent 5% of all hospitalizations in women of childbearing age. Hospitalizations for abortions in Brazil reduced 0.76 in the period; this trend occurred in 19 Brazilian FUs and all regions, except the South (stable). There was a significant reduction (p<0.001) in hospitalizations for spontaneous abortion and in admissions for abortion in women aged 20 to 39 years.

**Conclusion:**

A temporal trend of reduction in the hospitalizations due to abortion in Brazil was found, variating according to characteristics of the woman, FU and region of residence.

## Resumen

**Objetivo:**

Analizar la tendencia temporal de las hospitalizaciones por aborto en Brasil de 2008-2018, según región y unidad federativas (UF).

**Métodos:**

Estudio ecológico con datos sobre hospitalizaciones por aborto de mujeres en edad fértil del Sistema de Información Hospitalaria/ Sistema Único de Salud (SIH/SUS). Las tasas se calcularon según las características de la mujer y la tendencia se evaluó mediante regresión lineal generalizada de Prais-Winsten.

**Resultados:**

Las 2.258.104 hospitalizaciones por aborto representan el 5% de todas las hospitalizaciones en mujeres en edad fértil. Hubo una reducción significativa de 0,76 puntos porcentuales por año; esta tendencia ocurrió en 19 UFs brasileñas y en todas las regiones, excepto en el Sur (estable). Hubo una reducción significativa (p<0,001) en las hospitalizaciones por aborto espontáneo y en mujeres de 20 a 39 años.

**Conclusión:**

El estudio mostró una tendencia a reducir las hospitalizaciones por aborto en Brasil, con variaciones según características de la mujer, UF y región de residencia.

## INTRODUÇÃO

**Figure:**
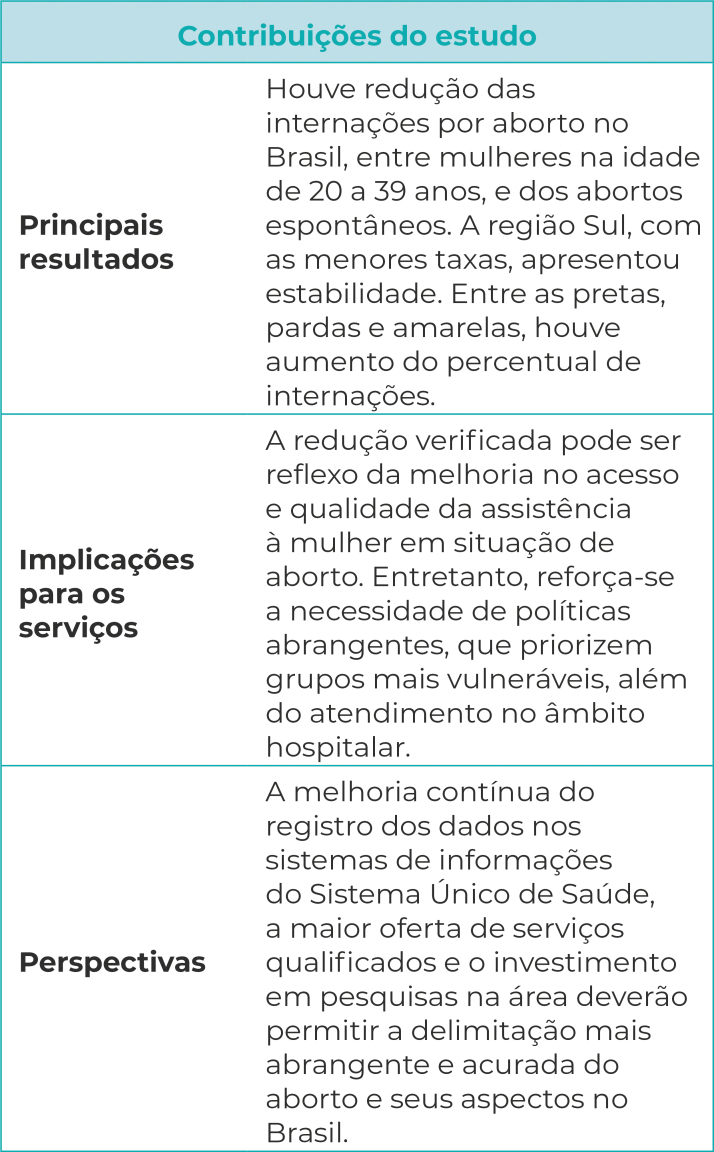

O aborto é uma intercorrência obstétrica em que há interrupção da gestação antes de atingida a viabilidade fetal, ocorrendo, mais frequentemente, nas primeiras 12 semanas, e atingindo 15% a 25% de todas as gestações.^1^^,^^2^ Apesar de 70% dos abortos serem resolvidos espontaneamente, em até oito semanas, podem ser necessárias intervenções médicas ou cirúrgicas para prevenir possíveis complicações maternas, como hemorragia, infecção, choque hipovolêmico e até mesmo o óbito.^2^

Entre 2015 e 2019, ocorreram aproximadamente 73,3 milhões de abortos a cada ano, no mundo, segundo a Organização Mundial da Saúde (OMS).^3^ Desses, 45% foram considerados abortos inseguros, ou seja, realizados por pessoas sem habilidades e/ou em ambientes inapropriados, sendo responsáveis por 4,7% a 13% das mortes maternas a cada ano.^3^^,^^4^ Desde 1990, as taxas de aborto diminuíram significativamente, nos países de maior renda.^1^ Estima-se uma ocorrência de 5 a 7 milhões de internações por complicações de aborto em países de baixa-média renda.^3^^,^^5^ De acordo com um estudo realizado em 13 países em desenvolvimento da África, Ásia, América Latina e Caribe, considerando-se o período de 1989 a 2003, uma a cada quatro mulheres que se submetem a um aborto inseguro poderá ter sequelas, temporárias ou permanentes, que requeiram assistência médica.^3^^,^^5^

No Brasil, o aborto é um problema de saúde pública, dada sua ocorrência em patamares elevados, ao longo dos anos, e suas graves consequências para a saúde da mulher.^6^ Presume-se que cerca de 1 milhão de abortos sejam praticados a cada ano, no Brasil.^7^ A interrupção voluntária da gestação é isenta de pena, segundo o Código Penal brasileiro de 1940, quando não há outro meio para salvar a vida da gestante ou quando a concepção decorreu de estupro.^8^ Mais recentemente, em 2012, a anencefalia fetal foi incluída na lista de permissivos legais à realização de aborto.^9^ Contudo, para uma parcela importante dos casos de aborto, não há amparo legal, o que leva à prática do aborto inseguro. Estima-se que, anualmente, cerca de 230 mil mulheres sejam internadas pelo Sistema Único de Saúde (SUS) em decorrência de abortos inseguros.^10^

Apesar da impossibilidade de se estimar a real carga do aborto no país, os sistemas de informações em saúde disponibilizam dados úteis para a análise epidemiológica do tema, conforme demonstra estudo descritivo do cenário do aborto no Brasil a partir de dados oficiais até 2015.^6^ Complementarmente, o presente estudo pretende atualizar os dados existentes e analisar as internações por aborto segundo região e Unidade da Federação (UF) do Brasil, considerando-se também a idade, a raça/cor da pele e o tipo de aborto. Estes dados ajudarão a ampliar a compreensão do problema desde suas especificidades regionais.

O objetivo deste estudo foi analisar a série temporal das internações por aborto no Brasil, no período de 2008 a 2018, segundo região e UF do país.

## MÉTODOS

Realizou-se um estudo ecológico de séries temporais. Foram analisadas as internações por aborto registradas no Sistema de Informações Hospitalares do Sistema Único de Saúde (SIH/SUS), para todas as UFs do Brasil, no período de 2008 a 2018.

Segundo dados do Censo Demográfico de 2010, a população do país era de 190.755.799 habitantes, sendo o Sudeste a região mais populosa, com 42% da população total. São 53.669.289 mulheres em idade fértil (faixa etária de 15 a 49 anos),^11^ a maioria delas conta 15 anos ou mais (56%) e possui ensino fundamental completo ou maior escolaridade, com variação de 48% na região Nordeste a 60% nas regiões Sudeste e Centro-Oeste.^12^

Os dados do SIH/SUS são relativos a todas as internações hospitalares realizadas pelo SUS no território nacional. A população-alvo deste estudo foi captada dos registros de internação de mulheres em idade fértil (15 a 49 anos), referentes a todas as UFs brasileiras, entre 2008 e 2018, período definido de acordo com a disponibilidade de dados no momento do acesso ao sistema de informações, realizado de agosto a novembro de 2019. Considerou-se internação por aborto aquela que teve as seguintes causas registradas, segundo a Décima Revisão da Classificação Estatística Internacional de Doenças e Problemas Relacionados à Saúde (CID-10): Aborto espontâneo (O03); Aborto por razões médicas (O04); Outras gravidezes que terminam em aborto (O00-O02, O05-O08).

Foram considerados todos os registros de aborto encontrados no banco de dados do SIH/SUS, disponibilizados no sítio eletrônico do Departamento de Informática do Sistema Único de Saúde (Datasus), do Ministério da Saúde. Os dados populacionais foram captados do censo populacional do Instituto Brasileiro de Geografia e Estatística (IBGE) e de projeções intercensitárias para o período de 2008 a 2018, também disponibilizados pelo Datasus.

As variáveis estudadas foram: ano de ocorrência (2008 a 2018); macrorregião nacional (Norte; Nordeste; Sudeste; Sul; Centro-Oeste); UF de residência (Rondônia, Acre, Amazonas, Roraima, Pará, Amapá, Tocantins; Maranhão, Piauí, Ceará, Rio Grande do Norte, Paraíba, Pernambuco, Alagoas, Sergipe, Bahia; Minas Gerais, Espírito Santo, Rio de Janeiro, São Paulo; Paraná, Santa Catarina, Rio Grande do Sul; Mato Grosso do Sul, Mato Grosso, Goiás, Distrito Federal); faixa etária da mulher (em anos: 15 a 19; 20 a 29; 30 a 39; 40 a 49); sua raça/cor da pele (branca; preta; parda; amarela; indígena; sem informação); e a categoria registrada para o tipo de aborto (espontâneo; por razões médicas; outras gravidezes que terminam em aborto). Os dados ignorados foram excluídos das tabulações específicas de cada variável.

 As taxas de hospitalização por aborto, de acordo com a faixa etária, UF de residência, tipo de aborto e ano do procedimento, foram calculadas pela divisão da soma das internações por aborto por todas as causas, pela população feminina em idade fértil no mesmo local e período, e o quociente obtido multiplicado por 10 mil mulheres da mesma faixa etária. Os dados relativos ao tipo de aborto e raça/cor da pele foram analisados por meio do cálculo da proporção de abortos em cada categoria, em relação ao total de casos no período 2008-2018.

Foi calculada a variação percentual das taxas, considerando-se os anos inicial e final da série, aplicando-se a seguinte fórmula:

[(taxa final - taxa inicial) / taxa inicial] x 100

Também foi calculada a taxa média do período estudado, por região e UF, e respectivos intervalos de confiança de 95% (IC_95%_).

A análise da série temporal foi realizada utilizando-se o modelo de regressão linear generalizada de Prais-Winsten com variância robusta; e a estatística de Durbin-Watson, para verificar a presença de autocorrelação serial. Foram avaliadas as taxas médias de cada série, o coeficiente de determinação das séries (R^2^) e a variação média anual dos valores das séries, esta dada pelo coeficiente β. A variável-resposta (Yi) foi a taxa de internação por aborto; e a variável explicativa (Xi), o ano de internação. O valor do coeficiente angular (β) positivo/negativo representou o aumento/decréscimo médio anual nas internações por aborto para cada ano observado. Foram considerados significativos os p-valores<0,05.

Por se tratar de estudo ecológico, com acesso a dados agregados e disponibilizados publicamente, o projeto do estudo não teve de ser submetido a um comitê de ética em pesquisa, embora tenha seguido as demais recomendações da Resolução do Conselho Nacional de Saúde (CNS) n^o^ 466, de 12 de dezembro de 2012.

## RESULTADOS

Foram registradas, no período de 2008 a 2018, 2.258.104 internações por aborto no Brasil, correspondendo a uma taxa média anual de 37,4 internações a cada 10 mil mulheres em idade fértil. A proporção das internações por aborto de mulheres em idade fértil foi de 5,2%, considerando-se o total de internações da população feminina da mesma faixa etária, para o mesmo período.

A análise temporal, de 2008 a 2018, indicou uma redução significativa das internações por aborto no Brasil, com uma variação média de 0,76 ponto percentual (p.p.) ao ano (Tabela 1). Essa redução foi observada em quase todas as regiões: a exceção coube à região Sul, onde se verificou estabilidade. As UFs das regiões Norte e Nordeste apresentaram as maiores taxas, com destaque para Roraima, Amapá e Acre, no Norte, e Sergipe e Bahia no Nordeste. Nestas regiões, também foram verificadas as maiores quedas nas taxas no período, maiores que 1,00 p.p. ao ano.

**Tabela 1:** - Taxa de internações por aborto entre mulheres de 15 a 49 anos de idade, segundo Unidade da Federação, Brasil, 2008-2018

Região/Unidade da Federação	Taxa de internação por aborto na população feminina em idade fértil^a^														
	2008	2009	2010	2011	2012	2013	2014	2015	2016	2017	2018	Média (IC_95%_^b^)	β^c^	p-valor	R^2 d^
Brasil	41,4	41,0	40,2	38,3	37,6	36,7	36,9	36,1	34,5	35,5	33,3	37,4 (35,6;39,1)	-0,76	<0,001	0,94
Norte	57,7	55,8	55,4	52,5	50,5	47,6	45,6	44,8	43,0	45,4	42,7	49,2 (45,5;52,8)	-1,54	<0,001	0,98
Rondônia	34,0	44,4	42,4	43,8	42,0	40,6	42,5	38,6	38,0	38,9	40,3	40,5 (38,5;42,5)	0,06	0,912	0,01
Acre	79,5	73,6	87,0	79,5	80,2	67,1	62,8	67,1	63,9	66,4	58,5	71,4 (65,4;77,5)	-2,22	0,002	0,71
Amazonas	70,6	59,0	60,0	49,5	43,2	41,0	35,8	36,8	40,3	43,4	45,9	47,8 (40,4;55,2)	-12,50	0,009	0,66
Roraima	97,7	98,6	102,8	102,4	96,0	90,4	81,3	84,4	83,2	83,6	87,2	91,6 (86,1;97,1)	-1,27	0,083	0,91
Pará	48,6	48,7	46,8	44,9	45,2	43,4	41,9	41,8	38,7	41,7	38,2	43,6 (41,2;46,0)	-1,04	<0,001	0,99
Amapá	107,2	108,6	102,6	105,8	98,6	89,9	84,1	76,2	63,9	64,3	60,9	87,5 (74,9;99,9)	-4,94	<0,001	0,94
Tocantins	56,3	52,7	52,4	55,8	54,9	51,1	54,1	49,1	44,6	44,4	28,5	49,5 (44,0;54,9)	-2,54	0,067	0,76
Nordeste	50,9	50,0	48,6	45,5	44,4	42,7	43,4	42,5	39,1	41,1	39,2	44,3 (41,6;47,0)	-1,17	<0,001	0,96
Maranhão	41,7	41,1	42,6	42,8	43,5	44,6	43,7	44,0	39,2	43,3	42,9	42,7 (41,6;43,7)	0,07	0,634	0,11
Piauí	53,0	49,6	47,7	42,7	42,1	43,4	42,5	43,4	43,9	44,5	43,7	45,1 (42,8;47,5)	-3,69	0,022	0,46
Ceará	51,7	48,6	47,9	45,0	43,3	40,8	42,7	42,1	39,0	40,8	39,9	43,8 (41,1;46,5)	-1,14	0,002	0,96
Rio Grande do Norte	28,9	42,7	42,7	35,6	35,5	35,9	44,9	44,8	38,5	40,9	39,0	39,0 (35,8;42,3)	0,59	0,364	0,00
Paraíba	46,9	47,7	43,2	41,0	41,5	38,5	39,8	39,0	35,5	42,3	39,5	41,4 (38,9;43,8)	-0,79	0,040	0,80
Pernambuco	47,8	44,5	45,3	42,6	41,9	40,4	40,5	39,9	34,0	36,9	34,9	40,8 (37,9;43,7)	-1,22	<0,001	0,97
Alagoas	51,0	48,3	47,2	42,4	41,2	38,8	37,3	37,5	36,4	39,0	35,3	41,3 (37,7;44,9)	-1,54	0,002	0,95
Sergipe	69,1	63,6	61,4	56,0	53,0	48,7	47,8	50,0	41,0	39,8	37,4	51,6 (44,7;58,5)	-3,03	<0,001	0,96
Bahia	59,1	58,5	55,0	51,8	49,7	46,7	46,8	43,8	42,8	42,6	40,1	48,8 (44,4;53,2)	-1,92	<0,001	0,99
Sudeste	36,2	36,3	35,7	34,1	33,7	33,2	33,5	32,5	31,2	31,5	28,9	33,3 (31,8;34,9)	-0,66	<0,001	0,94
Minas Gerais	38,6	38,1	37,1	36,4	36,1	34,3	34,4	33,2	32,0	32,5	30,1	34,8 (32,9;36,6)	-0,79	<0,001	0,99
Espírito Santo	37,7	34,2	31,7	33,2	33,6	31,8	29,6	29,5	28,5	27,9	25,9	31,2 (28,9;33,5)	-0,99	<0,001	0,94
Rio de Janeiro	36,6	37,8	38,4	35,5	35,1	38,3	38,9	39,7	35,3	34,1	29,3	36,3 (34,3;38,2)	-0,67	0,227	0,80
São Paulo	34,7	35,0	34,3	32,7	32,1	31,0	31,4	29,9	29,6	30,5	28,5	31,8(30,3;33,3)	-0,63	<0,001	0,92
Sul	31,1	31,0	30,9	30,3	31,2	30,6	31,6	30,9	31,3	32,0	30,2	30,9 (30,6;31,4)	0,03	0,560	0,94
Paraná	27,6	28,5	29,0	29,7	30,1	30,3	31,4	31,0	31,4	33,4	32,2	30,4 (29,3;31,6)	0,49	<0,001	0,99
Santa Catarina	35,2	34,9	33,9	33,0	34,3	34,9	35,4	34,2	36,0	36,5	34,1	34,8 (34,1;35,4)	0,06	0,636	0,87
Rio Grande do Sul	32,2	31,2	31,0	29,2	30,3	28,2	29,2	28,7	28,2	27,7	25,5	29,2 (27,9;30,5)	-0,52	<0,001	0,87
Centro-Oeste	38,5	37,3	35,5	34,3	32,4	33,0	32,5	32,1	30,9	31,3	30,2	33,5 (31,7;35,3)	-0,81	<0,001	0,98
Mato Grosso do Sul	37,9	35,2	36,7	36,0	34,0	35,6	34,5	34,5	34,0	35,1	29,8	34,9 (33,5;36,2)	-0,44	0,020	0,50
Mato Grosso	35,1	33,7	32,8	33,2	32,9	34,7	34,7	37,4	34,0	36,2	38,6	34,9 (33,6;36,1)	0,37	0,105	0,73
Goiás	30,5	30,5	28,8	28,6	27,9	28,6	29,2	29,0	27,2	27,5	26,8	28,6 (27,8;29,4)	-0,32	0,001	0,96
Distrito Federal	59,9	57,6	51,8	46,1	40,2	38,2	35,3	31,4	33,1	31,0	29,4	41,3 (33,9;48,6)	-6,42	0,004	0,66

Fonte: Departamento de Informática do Sistema Único de Saúde (Datasus); adaptação dos autores (2008-2018).

a) Taxa por 10 mil mulheres em idade fértil; b) IC_95%_: intervalo de confiança de 95%; c) O coeficiente β da regressão expressa a variação anual média, em pontos percentuais, ao ano; d) R^2^: coeficiente de determinação das séries.

Em contraposição, todas as UFs das regiões Sul e Sudeste apresentaram taxas inferiores à média nacional; o mesmo foi observado para quase todas as UFs da região Centro-Oeste, onde a exceção coube ao Distrito Federal. No Sudeste, as maiores taxas foram verificadas no Rio de Janeiro; e no Sul, em Santa Catarina. No Centro-Oeste, Goiás apresentou as menores taxas quando comparado às demais UFs da região. Entre as 27 UFs, 19 apresentaram reduções percentuais significativas no período, sendo as maiores no estado do Amazonas (-12,50 p.p./ano), no Distrito Federal (-6,42 p.p./ano) e no estado do Amapá (-4,94 p.p./ano). Rondônia, Roraima, Tocantins, Maranhão, Rio Grande do Norte, Rio de Janeiro, Santa Catarina e Mato Grosso mantiveram as taxas estáveis no período.

**Figura 1:**
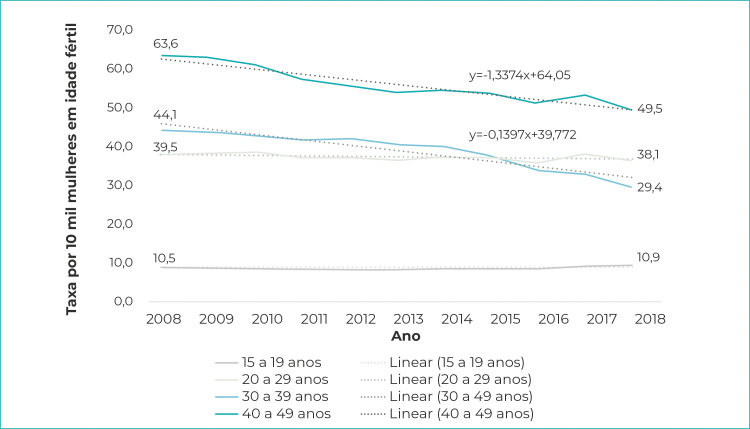
- Taxa de internações por aborto segundo faixa etária da população feminina, Brasil, 2008-2018

A avaliação por faixa etária indicou maior taxa de internações por aborto em mulheres na idade entre 20 e 29 anos (média anual de 56,0 casos/10 mil mulheres; IC_95%_ 52,9;59,1); no período analisado, houve redução significativa de internações por essa causa nessa faixa etária (de 63,6 a 49,5/10 mil; β=-1,34; R^2^=0,98), o que também foi observado para mulheres de 30 a 39 anos, embora com menor variação (de 39,5 a 38,1/10 mil; β=-0,14; R^2^=0,97). Entre adolescentes (15 a 19 anos) e mulheres a partir de 40 anos, as taxas de internações por aborto mostraram-se estáveis no período (Figura 1).

**Figura 2:**
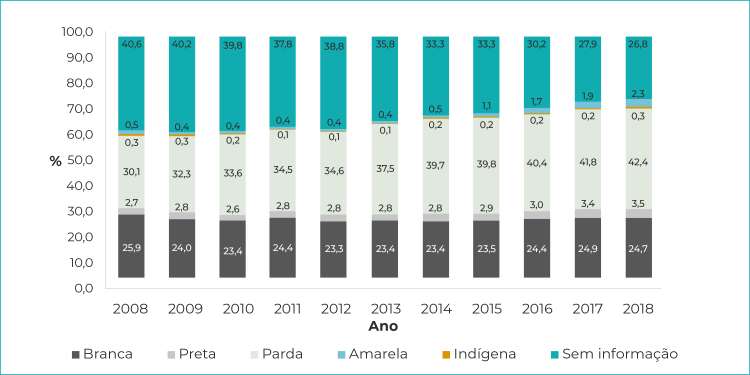
- Proporção de internações por aborto segundo raça/cor da pele, Brasil, 2008-2018

A avaliação por raça/cor da pele indicou 34,9% de dados ignorados, embora se tenha observado aumento no percentual de registros ao longo do período analisado (de 40,6% sem informação, em 2008, para 26,8% em 2018). Entre as mulheres brancas, houve uma pequena redução (de 25,9% em 2008, para 24,7% em 2018), enquanto as indígenas permaneceram com percentual de 0,3%. Para mulheres identificadas como pretas, pardas e amarelas, o percentual de internações aumentou (Figura 2).

Nota: Dada a pouca expressão dos registros de abortos nas categorias ‘amarela’ e ‘indígena’, a representação destes estratos de raça/cor da pele fica indistinguível, especialmente nos anos iniciais da série, quando os valores proporcionais são semelhantes.

**Figura 3:**
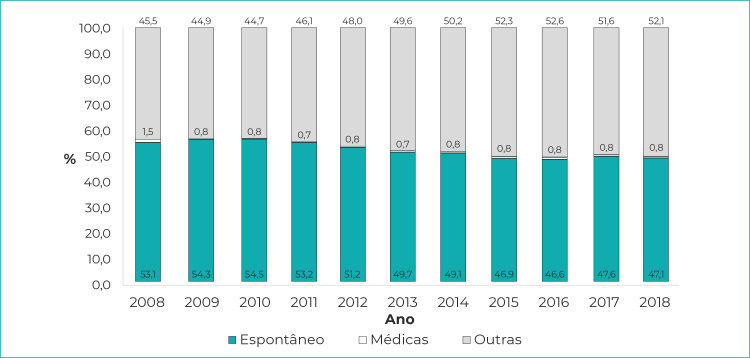
Proporção de internações segundo tipo de aborto registrado, em relação ao total de internações por aborto, Brasil, 2008-2018

Ao longo do período analisado, no que se refere ao tipo de aborto registrado, a categoria mais frequente foi ‘Espontâneo’, que totalizou 1.138.096 internações - ou 50,3% das internações por aborto. Seguiram-se as internações decorrentes de ‘Outras gravidezes que terminam em aborto’, que compreenderam 1.100.949 - ou 48,9% das internações. As internações cuja causa foi ‘Razões médicas’ corresponderam a 0,8% do total de internações por aborto (Figura 3). Observou-se redução significativa das internações por abortos espontâneos (β=-0,86; R^2^=0,85; p-valor<0,001), concomitantemente ao aumento de internações por outras gravidezes que terminam em aborto (β=0,89; R^2^=0,88; p-valor<0,001).

Nota: Dada a pouca expressão dos registros de abortos por razões médicas, os valores desta categoria são pouco perceptíveis.

## DISCUSSÃO

O aborto representou cerca de 5% do total das hospitalizações de mulheres em idade fértil no período de 2008 a 2018, com taxa média anual de 37,4 internações a cada 10 mil mulheres. Evidenciou-se redução nas internações por aborto no Brasil, sendo a maior queda verificada nas UFs das regiões Norte e Nordeste, as quais apresentaram, ainda assim, taxas superiores à verificada nacionalmente. Observou-se, também, redução das internações por abortos espontâneos concomitantemente ao aumento das internações por aborto devido a outras gravidezes que terminam em aborto.

Essa tendência de queda nas taxas de internação por aborto já se apresentara anteriormente, para a década 2000-2010, quando houve uma redução de 11% de internações hospitalares por essa causa.^13^ No entanto, somente uma parcela dos abortos requer internação hospitalar. O número total de abortos praticados no Brasil, dado a ser utilizado no cálculo da proporção de complicações/internações devidas a essa causa, é desconhecido. Um estudo nacional demonstrou que o aborto é um evento frequente na vida das mulheres brasileiras e que, ao final da vida reprodutiva, uma a cada cinco mulheres haviam realizado aborto no país.^14^

A disponibilidade de serviços é escassa para o atendimento do aborto conforme previsto em lei e, mesmo considerando-se o atendimento mais amplo das complicações pós-aborto, o estigma ligado ao aborto induzido representa uma limitação no acesso a esse serviço.^15^ Estudo conduzido com mulheres que receberam atendimento pós-aborto, em seis países latino-americanos, incluído o Brasil, concluiu: três em cada quatro mulheres referiram ansiedade e estresse durante a internação; 12,5% afirmaram não ter recebido explicações sobre autocuidado; e 12,8% relataram não ter sido concedida a elas, durante o atendimento, a oportunidade de perguntar sobre seus exames e tratamento.^16^

Entretanto, houve melhorias significativas na qualidade dos registros das informações em saúde do SUS, principalmente os do SIH/SUS, permitindo a tabulação de internações por procedimento ou diagnóstico relacionado ao aborto. Isto posto, é possível supor que as internações por aborto, efetivamente, estejam se reduzindo no Brasil, não sendo possível todavia afirmar, a partir desses dados, se está a haver uma queda na ocorrência de abortos.^6^ Existem evidências de importantes avanços na tecnologia do aborto medicamentoso, em nível mundial, fato que pode ter reduzido a necessidade de recorrer a práticas perigosas, como uso de agulhas de tricô, talos de vegetais ou produtos tóxicos, mesmo em contextos de ilegalidade.^3^^,^^17^

No Brasil, a Pesquisa Nacional do Aborto (PNA), realizada em 2010, revelou que 48% das mulheres realizaram o procedimento usando medicamento, supostamente o misoprostol: das 122 mulheres entrevistadas em profundidade, na âmbito da referida pesquisa, e que utilizaram o misoprostol como método único, 47% não precisaram ser internadas para finalizar o aborto.^18^ Em Portugal, estudo sobre as internações por aborto, desenvolvido no período de 2000 a 2014, demonstrou redução nas hospitalizações por aborto após sua legalização, ocorrida em 2007, além de um decréscimo nas admissões por urgência no mesmo período.^19^ Nos Estados Unidos, onde o aborto também foi descriminalizado e mais de 80% dos abortos são cirúrgicos, verificou-se, de 2003 a 2012, uma redução nas taxas de abortos cirúrgicos e aumento dos abortos por medicação.^20^ Estudo realizado a partir dos requerimentos de aborto ao sistema de saúde do estado da Califórnia, Estados Unidos, indicou que 28% das mulheres usaram medicação e 7,1% tiveram atendimento hospitalar.^21^ Dados do mesmo estudo, conduzido no Brasil e em mais cinco países latino-americanos, mostraram que a prevalência de complicações pós-aborto, entre moderadas e graves, foi de 53,8%, ilustrando que as complicações secundárias ao aborto seguem sendo um importante problema de saúde pública e configuram um persistente desafio para as políticas de saúde na América Latina.^16^

No Brasil especialmente, a maioria das grandes regiões nacionais apresentou redução das internações por aborto, mais expressiva no Norte do país. A região Sul, que já apresentava as menores taxas nacionais, mostrou estabilidade no período avaliado. As maiores taxas de internação por aborto foram encontradas nos estados do Norte e Nordeste, além do Distrito Federal. Apesar da tendência de redução, essas regiões ainda apresentam médias superiores à nacional. As reduções nas internações podem, em parte, resultar de políticas específicas de educação e atenção em saúde adotadas em cada localidade, além da estrutura de serviços de saúde disponível. Ainda, maiores índices de analfabetismo e dificuldade de acesso à informação e aos serviços de saúde seriam possíveis causas dessas diferenças entre regiões, e mesmo entre UFs de uma mesma região.^13^ Ser solteira, chegar ao serviço com idade gestacional maior que 13 semanas e com produto da concepção já tendo sido expulso (situações que, provavelmente, refletem a dificuldade de acesso e falta de informação em saúde) foram fatores estatisticamente associados à maior gravidade das complicações por aborto em um conjunto de países latino--americanos, Brasil entre eles.^16^

Como esperado, a faixa etária dos 20 aos 29 anos apresentou a maior taxa de internação por aborto, e menores taxas e estabilidade para mulheres acima de 39 anos. A PNA, ao analisar dados de 2010 e 2016, encontrou resultados similares, evidenciando que o pico de ocorrência do aborto acontece no centro do período reprodutivo, isto é, entre os 18 e os 29 anos,^14^ e em menor proporção nas mulheres a partir de 25 anos de idade. É importante ressaltar que a pesquisa foi baseada em um inquérito domiciliar, cuja amostra aleatória constituiu-se de mulheres alfabetizadas, residentes em área urbana e na faixa etária de 18 a 39 anos,^9^^,^^14^ com representatividade para além das hospitalizações decorrentes do aborto.

No que diz respeito à raça/cor da pele, a grande proporção de dados ignorados limitou uma análise detalhada dessa característica, como já fora verificado em estudo anterior realizado no Brasil.^6^ A PNA observou taxas de abortos mais elevadas entre mulheres amarelas, pardas e indígenas, comparadas às de raça/cor da pele branca. No entanto, alguns subgrupos têm tamanho pequeno, como indígenas e amarelas, o que pode ter afetado a precisão das medidas em relação aos grupos mais numerosos.^6^^,^^14^ Deve-se considerar que as minorias étnico-raciais compõem um grupo de maior vulnerabilidade, por questões socioeconômicas e menor acesso aos serviços de saúde, sendo portanto menos captadas pelo registro de internações hospitalares.^22^ Além disso, a PNA estudou os abortos não previstos em lei, diferentemente do presente estudo, cujo objeto de análise foram todos os casos de internação hospitalar.

A redução da proporção de internações por aborto sem informação sobre raça/cor da pele, ao longo do período estudado, foi acompanhada de um leve aumento na proporção de mulheres pretas e pardas, sugerindo que a melhoria da informação tenha ocorrido nesse estrato populacional. Essa provável migração de dados sem informação para a população negra indica que a invisibilidade desse grupo de mulheres mais vulneráveis parece ter-se reduzido gradativamente, ao longo dos tempos recentes. Comparando-se com dados atuais de mortalidade por aborto, por exemplo, são essas mulheres as que se encontram no grupo de maior risco: estudo de Cardoso et al.^6^ mostrou que mulheres de raça/cor da pele preta e indígena têm maior risco de óbito por aborto, assim como aquelas das regiões Norte, Nordeste e Centro-Oeste. Os estratos populacionais e regiões com maiores taxas de hospitalização por aborto, verificados neste estudo, são também aqueles que apresentam menor frequência de pré-natal adequado, segundo dados da Pesquisa Nacional de Saúde (PNS) de 2013,^23^ sugerindo relação entre essas variáveis, provavelmente vinculadas ao acesso aos serviços de saúde de maneira geral.

Quanto ao tipo de aborto, verificou-se que a proporção de internações por aborto espontâneo foi maior. Uma análise do cenário do aborto no país, com base em diferentes sistemas de informações, sugeriu que as internações pelo SUS referidas como ‘Aborto por razões médicas’ sejam uma forma de avaliar o acesso ao aborto legal no Brasil, ao encontrar, em média, 1.600 internações/ano entre 2008 e 2015.^6^ No presente estudo, a média de internações por essa causa, no período 2008-2018, foi de aproximadamente 1.900 internações/ano, possivelmente explicadas pela ampliação do número de hospitais dedicados à interrupção legal da gestação conforme preconiza o Ministério da Saúde, visando prestar um atendimento humanizado ao abortamento, assim como às pessoas em situação de violência sexual.^24^^,^^25^ Também é importante aventar a possibilidade de estar se realizando um diagnóstico inicial mais criterioso e, portanto, um melhor preenchimento da Autorização de Internação Hospitalar pelo SUS (AIH-SUS). Outrossim, o aumento da proporção dos registros com CID-10 ‘Outras gravidezes que terminam em aborto’ pode sinalizar um acréscimo das internações por abortos provocados no contexto ilegal.

Entre países com leis restritivas sobre aborto, caso do Brasil, quando não previstos em lei, os abortos são, muitas vezes, realizados sem as mínimas condições de segurança.^1^ Tal situação, além de não reduzir a ocorrência de abortos, eleva a mortalidade materna por essa causa, quando restringe dramaticamente o acesso ao aborto seguro.^3^^,^^26^ A interrupção voluntária de uma gravidez envolve conflitos morais, éticos e religiosos que, aliados à condenação social e reforçados pela ilegalidade, resultam em omissão do relato ou sua declaração como “espontâneo”.^6^^,^^13^ Assim, dentro da taxa de internações por aborto espontâneo, há de se considerar uma parcela desconhecida que, de fato, corresponde a abortos provocados.

O presente estudo apresenta limitações. Inicialmente, por utilizar uma base de dados secundários, ele pode ser influenciado por incompletudes e inconsistências de dados, não verificáveis. Além disso, a pesquisa considerou todas as gravidezes que terminam em aborto (CID-10: O00-O08), com base na lista de morbidades disponível no SIH/SUS, limitando sua comparação com estudos que excluíram os códigos O00-O02, referentes a situações clínicas de gravidez ectópica e doença trofoblástica. A grande proporção de dados ignorados no quesito raça/cor da pele, por sua vez, limitou o cálculo das taxas para essa variável; esse achado, em si, é importante porque mostra a necessidade de melhorar o registro da variável nos sistemas de informações. Entretanto, houve redução da proporção de dados ignorados no período analisado, coincidindo com aumento dos registros de mulheres de raça/cor da pele preta e parda.

Isto posto, salienta-se que avaliar a magnitude do aborto no Brasil por meio das internações vinculadas a tal condição, embora revele apenas parte da realidade, permite mensurar uma parcela do problema a demandar cuidados no ambiente hospitalar. Para que seja possível obter um panorama mais abrangente e acurado, são necessários investimentos em pesquisa e aprimoramento contínuo dos dados existentes.

Este estudo observou uma tendência de redução nas internações por aborto no país, no período de 2008 a 2018. As maiores quedas nessas internações aconteceram nas regiões Norte e Nordeste, entre mulheres da faixa etária dos 20 aos 29 anos, e para abortos registrados como espontâneos. Foi possível avaliar os dados das UFs individualmente e, assim, perceber disparidades, inclusive internas a cada região. Também foram observadas desigualdades segundo raça/cor da pele, sugerindo maior vulnerabilidade das mulheres pretas e indígenas. Enquanto o aborto for ilegal no Brasil, os registros oficiais permitirão, no máximo, realizar estimativas e levantar hipóteses, fundamentais para melhorar a compreensão do problema e, consequentemente, buscar produzir evidências possíveis em um contexto de ilegalidade. Dessa forma, o decréscimo das internações por aborto pode se manter, sem que de fato represente uma redução da interrupção da gravidez de muitas mulheres que permanecem à margem do SUS.
