# Supplementary material for: Hospitalization due to abortion in Brazil, 2008-2018: an ecological time-series study
Source: Epidemiol Serv Saude. 2022 Feb 28;31(1):e2021341. [Article in Portuguese] doi: 10.1590/S1679-49742022000100017 (PMC11334442; doi:10.1590/S1679-49742022000100017)
Supplement: Translation

# Translation: Hospitalization due to abortion in Brazil, 2008-2018: an ecological time-series study

**Authors:** Maíra Dutra Uliana, Daniela Ferreira D’Agostini Marin, Maura Belomé da Silva, Camila Giugliani, Betine Pinto Moehlecke Iser

**Affiliations:** 1 Universidade do Sul de Santa Catarina, Faculdade de Medicina, Tubarão, SC, Brazil; 2 Universidade Federal do Rio Grande do Sul, Programa de Pós-Graduação em Epidemiologia, Porto Alegre, RS, Brazil; 3 Universidade do Sul de Santa Catarina, Programa de Pós-Graduação em Ciências da Saúde, Tubarão, SC, Brazil

**Keywords:** Abortion, Hospitalization, Women’s Health, Public Health, Time Series Studies

## Abstract

**Objective:**

To analyze the temporal trend of hospitalizations caused by abortion in Brazil between 2008-2018, according to region and federal units (FU).

**Methods:**

Ecological study concerning hospitalizations due to abortion of women of childbearing age, from Hospital Information System data. The rates were calculated according the characteristics of women. The trend was assessed by Prais-Winsten generalized linear regression.

**Results:**

The 2,258,104 hospitalizations due to abortion represent 5% of all hospitalizations in women of childbearing age. Hospitalizations for abortions in Brazil reduced 0.76 in the period; this trend occurred in 19 Brazilian FUs and all regions, except the South (stable). There was a significant reduction (p<0.001) in hospitalizations for spontaneous abortion and in admissions for abortion in women aged 20 to 39 years.

**Conclusion:**

A temporal trend of reduction in the hospitalizations due to abortion in Brazil was found, variating according to characteristics of the woman, FU and region of residence.

## INTRODUCTION

Abortion is an obstetric intercurrence in which pregnancy is interrupted before fetal viability is reached, occurring most frequently in the first 12 weeks, and affecting 15% to 25% of all pregnancies.^1^^,^^2^ Although 70% of abortions happen spontaneously within eight weeks, medical or surgical interventions may be required to prevent possible maternal complications, such as bleeding, infection, hypovolemic shock and even death.^2^

Between 2015 and 2019, approximately 73.3 million abortions occurred worldwide each year according to the World Health Organization (WHO).^3^ Forty-five percent of them were considered to be unsafe abortions, that is, performed by people without the necessary skills and/or in inappropriate settings, accounting for 4.7% to 13% of maternal deaths each year.^3^^,^^4^ Abortion rates have fallen significantly since 1990 in higher-income countries.^1^ An estimated 5 to 7 million hospitalizations due to abortion complications occur in low- and middle-income countries.^3^^,^^5^ According to a study conducted in 13 developing countries in Africa, Asia, Latin America and the Caribbean from 1989 to 2003, one in four women who undergo unsafe abortion may have temporary or permanent sequelae requiring medical care.^3^^,^^5^

In Brazil, abortion is a public health problem, given its occurrence at high levels over the years, as well as its serious consequences for women’s health.^6^ Around one million abortions are presumed to be performed every year in Brazil.^7^ Voluntary interruption of pregnancy is not a crime, according to the 1940 Brazilian Penal Code, when there is no other means to save the pregnant woman’s life or when conception resulted from rape.^8^ More recently, in 2012, fetal anencephaly was included on the list of legally authorized reasons for abortion.^9^ However, there is no legal authorization for a considerable proportion of abortion cases, thus leading to the practice of unsafe abortion. It is estimated that about 230,000 women are hospitalized every year in Brazilian National Health System (SUS) facilities due to unsafe abortions.^10^

Despite the impossibility of estimating the real burden of abortion in Brazil, health information systems provide useful data for epidemiological analysis of the subject, as shown in a descriptive study of the abortion scenario in Brazil based on official data as at 2015.^6^ Adding to this, our study aims to update existing data and analyze hospitalizations due to abortion according to Brazil’s regions and Federative Units, also taking into account age, race/skin color and type of abortion. These data will help broaden understanding of the problem according to its regional specificities.

The objective of this study was to analyze the temporal trend of hospitalizations due to abortion in Brazil between 2008-2018, according to the country’s regions and Federative Units.

## METHODS

We conducted an ecological time-series study. We analyzed hospitalizations due to abortion recorded on the Hospital Information System of the Brazilian National Health System (SIH/SUS) for all the Brazilian Federative Units between 2008 and 2018.

According to data from the Demographic Census, in 2010 Brazil had a population of 190,755,799 inhabitants, with the Southeast being the most populous region, accounting for 42% of the total population. There are 53,669,289 women of childbearing age (15 to 49 years old),^11^ the majority of them are aged 15 or more (56%) and have complete elementary education or higher levels of education, ranging from 48% in the Northeast region to 60% in the Southeast and Midwest regions.^12^

SIH/SUS data relate to all SUS hospitalizations within the national territory. This study’s target population was taken from the hospitalization records of women of childbearing age (15 to 49 years), for all Brazilian Federative Units between 2008 and 2018. This period was defined according to data availability at the time of accessing the information system, namely between August and November 2019. Hospitalization due to abortion was considered that which had the following recorded causes, according to the Tenth Revision of the International Statistical Classification of Diseases and Related Health Problems (ICD-10): spontaneous abortion (O03); complications following induced termination of pregnancy (O04) (abortion for medical reasons); other pregnancies with abortive outcome (O00-O02, O05-O08).

We considered all abortion records found on the SIH/SUS database, available on the Ministry of Health National Health System Information Technology Department (DATASUS) website. Population data were taken from the Brazilian Institute of Geography and Statistics (IBGE) population census and its intercensal projections for the period 2008 to 2018, also available from DATASUS.

The variables studied were: year of occurrence (2008-2018); Brazilian macro-region (North; Northeast; Southeast; South; Midwest); Federative Unit of residence (Rondônia, Acre, Amazonas, Roraima, Pará, Amapá, Tocantins; Maranhão, Piauí, Ceará, Rio Grande do Norte, Paraíba, Pernambuco, Alagoas, Sergipe, Bahia; Minas Gerais, Espírito Santo, Rio de Janeiro, São Paulo; Paraná, Santa Catarina, Rio Grande do Sul; Mato Grosso do Sul, Mato Grosso, Goiás, Federal District); maternal age group (in years: 15-19; 20-29; 30-39; 40-49); maternal race/skin color (white; black; brown; yellow; indigenous; no information); and type of abortion category recorded (spontaneous; for medical reasons; other pregnancies with abortive outcome). Unknown data were excluded from the specific tabulations of each variable.

 The rates of hospitalization due to abortion, according to age group, Federative Unit of residence, type of abortion and year of abortion were calculated by dividing the sum of hospitalizations due to abortion for all causes by the female population of childbearing age in the same place and period, and the quotient obtained was multiplied by 10,000 women of the same age group. Data regarding type of abortion and race/skin color were analyzed by calculating the proportion of abortions in each category, in relation to the total number of cases in the period 2008-2018.

Percentage variation in the rates was calculated, taking the initial and final years of the series, by applying the following formula:

[(final rate - initial rate) / initial rate] x 100

The mean rate for the period studied was also calculated, by region and Federative Unit, as well as the respective 95% confidence intervals (95%CI).

The time series analysis was performed using the Prais-Winsten generalized linear regression model with robust variance; as well as the Durbin-Watson statistic, to check for the presence of serial autocorrelation. The mean rates of each series, the coefficient of determination of series (R2) and the mean annual variation of the values of the series were evaluated, whereby the latter were given by the β coefficient. The response variable (Yi) was the rate of hospitalization due to abortion; while the explanatory variable (Xi) was the year of hospitalization. The positive/negative slope coefficient value (β) represented the mean annual increase/decrease in hospitalizations due to abortion for each year observed. P-values <0.05 were considered significant.

Due to its being an ecological study, with access to aggregated and publicly available data, the study project did not have to be submitted to a research ethics committee, although it did follow the remaining recommendations of National Health Council Resolution No. 466, dated December 12, 2012. 

## RESULTS

Between 2008 and 2018, 2,258,104 hospitalizations due to abortion were recorded in Brazil, corresponding to a mean annual rate of 37.4 hospitalizations per 10,000 women of childbearing age. The proportion of hospitalizations due to abortion among women of childbearing age was 5.2%, considering total hospitalizations of the female population of the same age group, for the same period.

The temporal analysis, from 2008 to 2018, indicated a significant reduction in hospitalizations due to abortion in Brazil, with mean variation of 0.76 percentage point (p.p.) per year (Table 1). This reduction was seen in almost all of Brazil’s regions, except the Southern region which remained stable. The Federative Units in the North and Northeast regions had the highest rates, especially Roraima, Amapá and Acre, in the North, and Sergipe and Bahia in the Northeast. The biggest falls in the rates in the period, greater than 1.00 p.p. a year, also occurred in those regions.

**Table 1:** - Rate of hospitalization due to abortion among women aged 15-49, by Federative Unit, Brazil, 2008-2018

Region/ Federative Unit	Rate of hospitalization due to abortion in females of childbearing age^a^														
	2008	2009	2010	2011	2012	2013	2014	2015	2016	2017	2018	Mean (95%CI^b^)	β^c^	p-value	R^2 d^
Brazil	41.4	41.0	40.2	38.3	37.6	36.7	36.9	36.1	34.5	35.5	33.3	37.4 (35.6;39.1)	-0.76	<0.001	0.94
North	57.7	55.8	55.4	52.5	50.5	47.6	45.6	44.8	43.0	45.4	42.7	49.2 (45.5;52.8)	-1.54	<0.001	0.98
Rondônia	34.0	44.4	42.4	43.8	42.0	40.6	42.5	38.6	38.0	38.9	40.3	40.5 (38.5;42.5)	0.06	0.912	0.01
Acre	79.5	73.6	87.0	79.5	80.2	67.1	62.8	67.1	63.9	66.4	58.5	71.4 (65.4;77.5)	-2.22	0.002	0.71
Amazonas	70.6	59.0	60.0	49.5	43.2	41.0	35.8	36.8	40.3	43.4	45.9	47.8 (40.4;55.2)	-12.50	0.009	0.66
Roraima	97.7	98.6	102.8	102.4	96.0	90.4	81.3	84.4	83.2	83.6	87.2	91.6 (86.1;97.1)	-1.27	0.083	0.91
Pará	48.6	48.7	46.8	44.9	45.2	43.4	41.9	41.8	38.7	41.7	38.2	43.6 (41.2;46.0)	-1.04	<0.001	0.99
Amapá	107.2	108.6	102.6	105.8	98.6	89.9	84.1	76.2	63.9	64.3	60.9	87.5 (74.9;99.9)	-4.94	<0.001	0.94
Tocantins	56.3	52.7	52.4	55.8	54.9	51.1	54.1	49.1	44.6	44.4	28.5	49.5 (44.0;54.9)	-2.54	0.067	0.76
Northeast	50.9	50.0	48.6	45.5	44.4	42.7	43.4	42.5	39.1	41.1	39.2	44.3 (41.6;47.0)	-1.17	<0.001	0.96
Maranhão	41.7	41.1	42.6	42.8	43.5	44.6	43.7	44.0	39.2	43.3	42.9	42.7 (41.6;43.7)	0.07	0.634	0.11
Piauí	53.0	49.6	47.7	42.7	42.1	43.4	42.5	43.4	43.9	44.5	43.7	45.1 (42.8;47.5)	-3.69	0.022	0.46
Ceará	51.7	48.6	47.9	45.0	43.3	40.8	42.7	42.1	39.0	40.8	39.9	43.8 (41.1;46.5)	-1.14	0.002	0.96
Rio Grande do Norte	28.9	42.7	42.7	35.6	35.5	35.9	44.9	44.8	38.5	40.9	39.0	39.0 (35.8;42.3)	0.59	0.364	0.00
Paraíba	46.9	47.7	43.2	41.0	41.5	38.5	39.8	39.0	35.5	42.3	39.5	41.4 (38.9;43.8)	-0.79	0.040	0.80
Pernambuco	47.8	44.5	45.3	42.6	41.9	40.4	40.5	39.9	34.0	36.9	34.9	40.8 (37.9;43.7)	-1.22	<0.001	0.97
Alagoas	51.0	48.3	47.2	42.4	41.2	38.8	37.3	37.5	36.4	39.0	35.3	41.3 (37.7;44.9)	-1.54	0.002	0.95
Sergipe	69.1	63.6	61.4	56.0	53.0	48.7	47.8	50.0	41.0	39.8	37.4	51.6 (44.7;58.5)	-3.03	<0.001	0.96
Bahia	59.1	58.5	55.0	51.8	49.7	46.7	46.8	43.8	42.8	42.6	40.1	48.8 (44.4;53.2)	-1.92	<0.001	0.99
Southeast	36.2	36.3	35.7	34.1	33.7	33.2	33.5	32.5	31.2	31.5	28.9	33.3 (31.8;34.9)	-0.66	<0.001	0.94
Minas Gerais	38.6	38.1	37.1	36.4	36.1	34.3	34.4	33.2	32.0	32.5	30.1	34.8 (32.9;36.6)	-0.79	<0.001	0.99
Espírito Santo	37.7	34.2	31.7	33.2	33.6	31.8	29.6	29.5	28.5	27.9	25.9	31.2 (28.9;33.5)	-0.99	<0.001	0.94
Rio de Janeiro	36.6	37.8	38.4	35.5	35.1	38.3	38.9	39.7	35.3	34.1	29.3	36.3 (34.3;38.2)	-0.67	0.227	0.80
São Paulo	34.7	35.0	34.3	32.7	32.1	31.0	31.4	29.9	29.6	30.5	28.5	31.8(30.3;33.3)	-0.63	<0.001	0.92
South	31.1	31.0	30.9	30.3	31.2	30.6	31.6	30.9	31.3	32.0	30.2	30.9 (30.6;31.4)	0.03	0.560	0.94
Paraná	27.6	28.5	29.0	29.7	30.1	30.3	31.4	31.0	31.4	33.4	32.2	30.4 (29.3;31.6)	0.49	<0.001	0.99
Santa Catarina	35.2	34.9	33.9	33.0	34.3	34.9	35.4	34.2	36.0	36.5	34.1	34.8 (34.1;35.4)	0.06	0.636	0.87
Rio Grande do Sul	32.2	31.2	31.0	29.2	30.3	28.2	29.2	28.7	28.2	27.7	25.5	29.2 (27.9;30.5)	-0.52	<0.001	0.87
Midwest	38.5	37.3	35.5	34.3	32.4	33.0	32.5	32.1	30.9	31.3	30.2	33.5 (31.7;35.3)	-0.81	<0.001	0.98
Mato Grosso do Sul	37.9	35.2	36.7	36.0	34.0	35.6	34.5	34.5	34.0	35.1	29.8	34.9 (33.5;36.2)	-0.44	0.020	0.50
Mato Grosso	35.1	33.7	32.8	33.2	32.9	34.7	34.7	37.4	34.0	36.2	38.6	34.9 (33.6;36.1)	0.37	0.105	0.73
Goiás	30.5	30.5	28.8	28.6	27.9	28.6	29.2	29.0	27.2	27.5	26.8	28.6 (27.8;29.4)	-0.32	0.001	0.96
Distrito Federal	59.9	57.6	51.8	46.1	40.2	38.2	35.3	31.4	33.1	31.0	29.4	41.3 (33.9;48.6)	-6.42	0.004	0.66

Source: Brazilian National Health System Information Technology Department (DATASUS); adapted by the authors (2008-2018).

a) Rate per 10,000 women of childbearing age; b) 95%CI: 95% confidence interval; c) The β regression coefficient expresses mean annual change, in percentage point, per annum; d) R^2^: coefficient of determination of series.

On the other hand, all the Federative Units in the South and Southeast had rates below the national average; the same was found for almost all the Federative Units in the Midwest region, with the exception of the Federal District. In the Southeast, the highest rates were found in Rio de Janeiro; while in the South they were found in Santa Catarina. In the Midwest, Goiás had the lowest rates when compared to the other Federative Units in the region. Among the 27 Federative Units, 19 had significant percentage reductions in the period, the largest being in the state of Amazonas (-12.50 p.p./year), in the Federal District (-6.42 p.p./year) and in the state of Amapá (-4.94 p.p./year). The rates in Rondônia, Roraima, Tocantins, Maranhão, Rio Grande do Norte, Rio de Janeiro, Santa Catarina and Mato Grosso remained stable in the period.

Analysis by age group indicated a higher rate of hospitalizations due to abortion in women aged 20 to 29 years (annual mean of 56.0 cases/10,000 women; 95%CI 52.9;59.1); In the period analyzed, there was a significant reduction in hospitalizations due to this cause in this age group (from 63.6 to 49.5/10,000; β=-1.34; R2=0.98), which was also found for women aged 30 to 39 years, although with less variation (from 39.5 to 38.1/10,000; β=-0.14; R2=0.97). Among adolescents (15 to 19 years old) and women aged 40 and over, the rates of hospitalizations due to abortion were stable in the period (Figure 1).

**Figure 1:** - Rate of hospitalization due to abortion by female population age group, Brazil, 2008-2018

Analysis according to race/skin color indicated 34.9% unknown data, although there was an increase in the percentage of these records over the period analyzed (from 40.6% with no information in 2008, to 26.8% in 2018). Among women of white race/skin color there was a small reduction (from 25.9% in 2008, to 24.7% in 2018), while the percentage remained stable at 0.3% among indigenous women. In the case of women identified as being of black, brown and yellow race/skin color, percentage hospitalizations increased (Figure 2).

**Figure 2:** - Proportion of hospitalizations due to abortion, by race/skin color, Brazil, 2008-2018

Note: Given the low expression of abortion records in the ‘yellow’ and ‘indigenous’ categories, representation of these race/skin color strata is indistinguishable, especially in the first years of the series, when the proportional values are similar.

Regarding the type of abortion recorded, over the period analyzed the most frequent category was ‘Spontaneous’, totaling 1,138,096 hospitalizations - or 50.3% of hospitalizations due to abortion. This was followed by ‘Other pregnancies with abortive outcome’, which accounted for 1,100,949 - or 48.9% of hospitalizations. Hospitalizations caused by ‘Medical reasons’ corresponded to 0.8% of total hospitalizations due to abortion (Figure 3). There was a significant decrease in hospitalizations due to spontaneous abortions (β=-0.86; R2=0.85; p-value<0.001), concomitant with an increase in hospitalizations due to other pregnancies with abortive outcome (β=0.89; R2=0.88; p-value<0.001).

**Figure 3:** - Proportion of hospitalizations by type of abortion recorded, in relation to total hospitalizations due to abortion, Brazil, 2008-2018

## DISCUSSION

Abortion accounted for around 5% of total hospitalizations of women of childbearing age in the period from 2008 to 2018, with a mean annual rate of 37.4 hospitalizations per 10,000 women. There was a reduction in hospitalizations due to abortion in Brazil, with the largest decrease seen in the Federative Units of the North and Northeast regions, although their rates were still higher than the national rate. There was also a reduction in hospitalizations due to spontaneous abortions concomitant with an increase in hospitalizations due to other pregnancies with abortive outcome.

This downward trend in rates of hospitalization due to abortion had already been shown previously, for the decade 2000-2010, when there was an 11% reduction in hospitalizations due to this cause.^13^ However, not all abortions require hospitalization. The total number of abortions performed in Brazil, which would be the number to be used to calculate the proportion of complications/ hospitalizations due to this cause, is unknown. A national study has shown that abortion is a frequent event in the lives of Brazilian women and that at the end of their reproductive life, one in five women had had an abortion in Brazil.^14^

Availability of services for abortion care as provided by law is scarce and, even considering broader care for post-abortion complications, the stigma attached to induced abortion represents a limitation in accessing this service.^15^ A study conducted with women who received post-abortion care in six Latin American countries, including Brazil, concluded that three out of four women reported anxiety and stress during hospitalization; 12.5% reported not having received explanation about self-care; and 12.8% reported not having been given the opportunity to ask about their exams and treatment during care.^16^

However, there have been significant improvements in the quality of SUS health information records, especially those held on the SIH/SUS system, allowing hospitalizations to be tabulated by procedure or diagnosis related to abortion. That said, it is possible to assume that hospitalizations due to abortion are effectively reducing in Brazil, but it is not possible to affirm, based on these data, whether there is a decrease in the occurrence of abortions.^6^ There is evidence of important progress with medication abortion technology worldwide, which may have reduced the need for dangerous practices, such as using knitting needles, vegetable stalks or toxic products, even in contexts of illegality.^3^^,^^17^

In Brazil, the 2010 National Abortion Survey revealed that 48% of women had undergone abortion using medication, presumably misoprostol: of the 122 women interviewed in depth for the survey who used misoprostol as the sole method, 47% did not need to be hospitalized in order to complete their abortion.^18^ In Portugal, a study carried out between 2000 and 2014 on hospitalizations due to abortion showed a reduction in hospitalizations due to abortion after it was legalized in 2007, as well as a decrease in emergency admissions in the same period.^19^ In the United States, where abortion has also been decriminalized and more than 80% of abortions are surgical, between 2003 and 2012 there was a decrease in surgical abortion rates and an increase in medication abortions.^20^ A study of requests for abortion made to the California health care system indicated that 28% of women used medication and 7.1% had hospital care.^21^ Data from the study conducted in Brazil and in five other Latin American countries, mentioned above, found 53.8% prevalence of moderate to severe post-abortion complications, illustrating that complications secondary to abortion remain an important public health problem and represent a persistent challenge for health policies in Latin America.^16^

In Brazil in particular, most of the country’s major regions showed a reduction in hospitalizations due to abortion, most significantly in the Northern region. The Southern region, which already had the lowest national rates, showed stability in the period evaluated. The highest rates of hospitalization due to abortion were found in the Northern and Northeastern states, besides the Federal District. Despite the downward trend, the average rates in these regions are still above the national average. The reductions in hospitalizations may in part be the result of specific health education and care policies adopted in each location, in addition to available health service structure. Moreover, higher illiteracy rates and difficulty in accessing information and health services could be possible causes of these differences between regions, and even between Federative Units within the same region.^13^ Being a single woman, arriving at a health service with gestational age greater than 13 weeks and with the product of conception already having been expelled (situations that probably reflect difficulty of access and lack of health information) were factors statistically associated with greater severity of abortion complications in a set of Latin American countries including Brazil.^16^

As expected, the highest rate of hospitalization due to abortion was found in the 20-29 year old age group, while lower rates and stability were found for women over 39 years old. Data from 2010 to 2016 analyzed by the National Abortion Survey showed similar results, providing evidence that the peak in the occurrence of abortion happens in the middle of the reproductive period, that is, among women between 18 and 29 years old,^14^ and to a lesser extent among women aged 25 and over. It is important to emphasize that this information was based on a household survey, using a random sample of literate women, living in urban areas and aged 18 to 39 years old,^9^^,^^14^ with representativeness that went beyond abortion-related hospitalizations.

With regard to race/skin color, the large proportion of unknown data limited a detailed analysis of this characteristic, similar to the findings of a previous study conducted in Brazil.^6^ The National Abortion Survey found higher abortion rates among women of yellow, brown and indigenous race/skin color compared to those of white skin race/color. However, some subgroups are small in size, such as women of indigenous and yellow race/skin color, which may have affected the accuracy of these measurements in relation to larger groups.^6^^,^^14^ It should be taken into consideration that racial-ethnic minorities comprise a group that is more vulnerable, due to socioeconomic issues and less access to health services, and therefore appear less in hospital admission records.^22^ In addition, the National Abortion Survey studied abortions not provided for by law, unlike the present study, which had as its object of analysis all abortion cases involving hospitalization.

The reduction in the proportion of hospitalizations due to abortion with no information on race/skin color over the period studied was accompanied by a slight increase in the proportion of women of black and brown race/skin color, suggesting that information has improved in relation to this population stratum. This probable migration of data lacking information to people of black race/skin color indicates that the invisibility of this most vulnerable group of women appears to have reduced gradually more recently. When comparing current data on mortality due to abortion, for example, these are the women who are in the highest risk group: a study conducted by Cardoso et al.^6^ showed that women of black and indigenous race/skin color are at greater risk of death due to abortion, as well as those from the North, Northeast and Midwest regions. The population strata and regions with higher rates of hospitalization due to abortion, as found in this study, are also those with lower frequency of adequate prenatal care, according to data from the 2013 National Health Survey,^23^ suggesting a relationship between these variables, probably linked to access to health services in general.

Regarding type of abortion, the proportion of hospitalizations due to spontaneous abortion was found to be higher. An analysis of the abortion scenario in Brazil, based on different information systems, suggested that SUS hospitalizations referred to as ‘Abortion for medical reasons’ is a way of assessing access to legal abortion in Brazil and found 1,600 hospitalizations/year between 2008 and 2015 on average.^6^ Our study found approximately 1,900 hospitalizations/year on average for this cause between 2008 and 2018, possibly explained by the expansion of the number of hospitals dedicated to legal termination of pregnancy as recommended by the Ministry of Health, aiming to provide humanized abortion care, as well as care for people in situations of sexual violence.^24^^,^^25^ It is also important to consider the possibility that a more careful initial diagnosis may be being made, therefore resulting in better filling out of the SUS Hospital Admission Authorization form. On the other hand, the increase in the proportion of records categorized under ICD-10 ‘Other pregnancies with abortive outcome’ can signal an increase in hospitalizations due to abortions induced illegally.

Among countries with restrictive laws on abortion, as is the case of Brazil, when not provided for by law, abortions are often performed without minimum safety conditions.^1^ This situation, besides not reducing the occurrence of abortions, increases maternal mortality from this cause, by dramatically restricting access to safe abortion.^3^^,^^26^ Voluntary interruption of pregnancy involves moral, ethical and religious conflicts which, combined with social condemnation and reinforced by illegality, result in it not being reported or it being reported as “spontaneous”.^6^^,^^13^ Thus, within the rate of hospitalizations due to spontaneous abortion, it must be recognized that an unknown part of them does in fact correspond to induced abortions.

This study has limitations. Initially, because it has used a secondary database, it may be influenced by unverifiable incompleteness and data inconsistencies. In addition, the study considered all pregnancies with abortive outcome (CID-10: O00-O08), based on the list of morbidities available on the SIH/SUS system, thus limiting its comparison with studies that excluded codes O00-O02, referring to clinical situations of ectopic pregnancy and trophoblastic disease. In turn, the large proportion of unknown data on race/skin color limited the calculation of rates for this variable. This finding, in itself, is important because it shows the need to improve the recording of this variable on the information systems. However, there was a reduction in the proportion of unknown data in the period analyzed, coinciding with an increase in the recording of women of black and brown skin color.

That said, it should be highlighted that although it only reveals part of the reality, assessing the magnitude of abortion in Brazil based on data on hospitalizations due to abortion enables measurement of part of the problem that requires attention in the hospital environment. In order for a more comprehensive and accurate picture to be obtained, investments in research and continuous enhancement of existing data are needed.

This study found a downward trend in hospitalizations due to abortion in Brazil between 2008 and 2018. The largest decreases in these hospitalizations occurred in the North and Northeast regions, among women aged 20-29 years, and in relation to abortions recorded as being spontaneous. We were able to assess the data from each Federative Unit individually and thus note disparities, including those within each region of the country. Inequalities were also found in relation to race/skin color, suggesting greater vulnerability of women of black and indigenous race/skin color. For as long as abortion remains illegal in Brazil, the official records will at most allow us to make estimates and hypotheses which, even so, are fundamental for improving understanding of the problem and, consequently, for attempting to produce as much evidence as possible in a context of illegality. As such, the decrease in hospitalizations due to abortion may continue, without actually representing a reduction in the interruption of the pregnancies of many women who remain on the margins of the SUS.
